# Anthracyclines-Induced Cardiac Dysfunction: What Every Clinician Should Know

**DOI:** 10.31083/j.rcm2405148

**Published:** 2023-05-18

**Authors:** Armando Ferrera, Vincenzo Fiorentini, Simone Reale, Giorgio Solfanelli, Giacomo Tini, Emanuele Barbato, Massimo Volpe, Allegra Battistoni

**Affiliations:** ^1^Clinical and Molecular Medicine Department, Sapienza University of Rome, 00198 Rome, Italy; ^2^IRCCS San Raffaele, 00163 Rome, Italy

**Keywords:** anthracyclines-induced cardiotoxicity, drug-induced heart failure, anthracyclines, cardio-oncology, chemotherapy, cardiotoxicity

## Abstract

Chemotherapies have changed the prognosis of patients affected by cancer over the last 20 years, with a significant increase in survival rates. However, they can cause serious adverse effects that may limit their use. In particular, anthracyclines, widely used to treat both hematologic cancers and solid cancers, may cause cardiac toxicity, leading to the development of heart failure in some cases. This review aims to explore current evidence with regards to anthracyclines’ cardiotoxicity, with particular focus on the classifications and underlying molecular mechanisms, in order to provide an overview on the current methods of its diagnosis, treatment, and prevention. An attentive approach and a prompt management of patients undergoing treatment with anthracyclines is imperative to avoid preventable antineoplastic drug discontinuation and is conducive to improving both short-term and long-term cardiovascular morbidity and mortality.

## 1. Introduction

Heart failure (HF) is a clinical syndrome consisting of typical symptoms, such as breathlessness and fatigue, and signs, like elevated jugular venous pressure, pulmonary crackles, and peripheral oedema. It is caused by a structural and/or functional abnormality of the heart that results in elevated intracardiac pressures and/or inadequate cardiac output at rest and/or during exercise [1]. Nowadays, the incidence of HF in Europe is about 3/1000 person-years [2] whilst its increasing prevalence has reached about 1–2% of adults [3]. Despite improvements in HF treatment, mortality rate is still high (67% within 5 years from the diagnosis) [4]. The main causes of HF are coronary artery disease, hypertension, valve disease, arrhythmias, cardiomyopathies, diabetes, congenital heart disease, infectious diseases, and drugs [1]. Drug-induced HF is emerging as a potentially preventable form, with cytostatic agents, antidepressants, and immunomodulatory agents being the most common drugs correlated with HF. First recognized in 1960 with the introduction of anthracyclines as a treatment in oncological patients, drug-induced HF remains of interest today for its impact and severity [5]. Anthracyclines are cytostatic antibiotics derived from Streptomyces spp. and are used in the treatment of various types of cancers, as they have been the most important class of antitumor drugs available for years [6]. Doxorubicin (DOX) (also called adriamycin) is extensively used for the treatment of several solid tumors, such as soft tissue and bone sarcomas, breast, ovary, bladder, thyroid and lung cancer [7]. Daunorubicin and idarubicin are used for the treatment of hematologic cancers, such as leukemia [8, 9]. Epirubicin is indicated in the treatment of breast cancer both in metastatic disease and as adjuvant therapy in women with early breast cancer [10]. Their anticancer activity depends on their ability to interact with DNA through different mechanisms, including topoisomerase II inhibition, DNA intercalation, and DNA strand breakage leading to cancer cell death. Anthracyclines may also inhibit polymerase activity, regulate gene expression, and cause damage to the DNA of cancer cells by producing reactive oxygen species (ROS) [11, 12].

In this review, we summarize the available literature on the adverse effects of anthracyclines on the heart with regards to the epidemiology and pathogenetic mechanisms of cardiac toxicity. Furthermore, we will also discuss the diagnostic workflow, the treatments available at present, and possible prevention strategies for this drug-related complication.

## 2. Methods

We comprehensively searched the literature for data on the epidemiology, molecular mechanisms, diagnostic workflow, therapies, and preventive strategies of anthracyclines-induced cardiotoxicity. We used “anthracyclines” or “doxorubicin” or “daunorubicin” or “idarubicin” or “epirubicin” and “cardiovascular prevention” or “cardiotoxicity” or “cardio-oncology” or “left ventricular dysfunction” or “heart failure” as search terms. Articles published from 1998 to 1st October 2022 in English on both PubMed and MEDLINE were included. Most recent and largest original articles and meta-analyses have been selected. Reviews, consensus papers and guidelines were included if relevant. A search across the references of selected reports helped to identify further additional relevant studies.

## 3. Epidemiology of Anthracyclines-Induced Cardiotoxicity

According to 2022 European Society of Cardiology (ESC) guidelines on cardio-oncology [13], anthracyclines-induced cardiotoxicity may be either symptomatic, when signs and symptoms of HF appear, or asymptomatic, if there is only a reduction in systolic left ventricular (LV) function parameters in absence of symptoms. It can be acute, early onset chronic, or late onset chronic [14]. When acute, it occurs usually after a single dose. This presentation accounts for <1% of patients undergoing treatment and is characterized by an early onset of symptoms of HF, usually presenting as a transient LV dysfunction. Chronic presentation can have an early-onset or late onset. The first represents the most common type, occurring within one year of treatment with a dilated-hypokinetic cardiomyopathy possibly progressively evolving towards HF. Late-onset chronic cardiotoxicity usually develop after years (a median of 7 years after treatment) with a clinical presentation similar to that of early-onset. The chronic forms of cardiotoxicity are considered irreversible with a poor prognosis [14]. There is discordant data regarding the incidence of cardiotoxicity. Most of the data derives from retrospective studies, with substantial variability in its reported incidence, depending on the its definition, the type and cumulative dose of anthracyclines, and patient age and comorbidities. A recent large meta-analysis of all studies involving at least 100 patients treated with anthracyclines found an overall incidence of 3.1% for clinical HF, with an incidence of 2.0% in those with breast cancer and 4.8% in those with lymphoma patients [15]. Subclinical cardiotoxicity was seen in 13.8% of overall patients, 10.3% of the subset with breast cancer and 19.8% of the subset with lymphoma patients. The incidence of HF correlated with increasing age and cumulative dose [15]. Accordingly, another report found congestive HF in 2–4%, subclinical LV dysfunction in around 10%, and cardiac biomarker rise in 30–35% of patients [16]. Cardinale *et al*. [14] conducted a prospective study involving 2625 patients, with a mean follow-up of more than 5 years, and showed an overall incidence of anthracyclines-induced cardiotoxicity of 9%, with 98% occurring within the first year after the completion of chemotherapy. Recently a study reported the incidence for cardiotoxicity in long term survivors of pediatric cancer as being 5.98%, after a mean follow-up period of 9 years [17]. Anthracyclines-related cardiotoxicity is dose-dependent and it is related to cumulative dose of drugs as indicated in Table 1 [18, 19].

**Table 1.** **Dosages of anthracyclines and incidence of left ventricular dysfunction**.

Dose of drug (mg/$m^{2}$)	Incidence of left ventricular dysfunction (%)
Doxorubicin 400	3–5
Doxorubicin 550	7–26
Doxorubicin 700	18–48
Epirubicin >900	0.9–11.4
Idarubicin >90	18

## 4. Pathogenesis of Anthracyclines-Induced Cardiotoxicity

Cardiomyocytes are vulnerable to anthracycline-induced toxicity and as such, LV systolic dysfunction is the most common cardiac adverse effect of anthracyclines [19]. Several mechanisms have been implicated in the pathophysiology of cardiotoxicity, including oxidative stress, inflammation, mitochondrial injury, apoptosis, calcium (${\mathrm{Ca}}^{2+}$) dysregulation, endoplasmic reticulum (ER) stress, increased fibrosis, and dysregulation of autophagy.

It has long been known that anthracyclines can cause a dose-dependent redox cycling with increased level of intracellular ROS [20]. The oxidative stress caused by the production of both ROS and reactive nitrogen species (RNS), via induction of nitric oxide synthase, seems to play a crucial role in the development of cardiotoxicity [19]. Indeed, DOX has a quinone moiety which facilitates electron transfer to oxygen molecules and other cellular redox enzymes (e.g., cytochrome P450 reductase, NADH dehydrogenase). Reduction of DOX produces the semiquinone radical, which re-oxidizes in the presence of O2 generating ROS that is associated with protein oxidation lipid peroxidation and DNA damage [20]. RNS can damage cardiomyocytes through nitration and inactivation of key enzymes in the heart, such as myofibrillar creatine kinase [21, 22]. Free iron also contributes to DOX-mediated oxidative stress due to the propagation of ROS formation [23].

There is a strong interplay between inflammation and oxidative stress, with both causing myocardial injury. Indeed, oxidative stress may stimulate an inflammatory response through activating nuclear factor kappa B (NF-κB), a redox-sensitive transcription factor [24]. DOX has shown to upregulate the levels of several inflammatory factors, including interleukin-1β, IL-6, IL-17, and tumor necrosis factor-alpha in the heart [25]. DOX-related oxidative stress might also activate Nucleotide-binding and oligomerization domain (NOD)-like receptor family pyrin domain-containing protein 3 (NLRP3) inflammasome, which is a regulator of the innate immune system [26]. Furthermore, the transient receptor potential ankyrin 1 (TRPA1) channel is activated by DOX to cause cardiotoxicity by promoting oxidative stress and inflammation [25]. Moreover, DOX has been proven to increase toll-like receptor 5 expression leading to increased inflammation [27].

Mitochondrial injury is also a hallmark of exposure to anthracyclines. Indeed, electrostatic binding between mitochondrial cardiolipin and DOX leads to disruption of the activity of complexes I, III, and IV in the electron transport chain (ETC). DOX accumulation in mitochondria is associated with enhanced production of ROS and RNS [28] followed by peroxidation of lipids and oxidative damage to DNA and proteins, resulting in mitochondrial DNA damage, loss of adenosine triphosphate (ATP) levels, peroxidation of cardiolipin and mitochondrial permeability transition [29]. The subsequent release of cytochrome C may trigger apoptosis of cardiac cells. In this setting, the role of nicotinamide adenine dinucleotide phosphate (NADPH) oxidase/ROS-mediated NF-κB-signaling cascade through the extracellular signal-regulated kinases 1 and 2 (ERK1/2) are fundamental in triggering DOX-mediated apoptosis [30, 31]. Indeed, activated ERKs phosphorylates p53 leading to cardiomyocyte apoptosis via downregulation of antiapoptotic B-cell lymphoma 2 (Bcl-2), upregulation of proapoptotic Bcl-2-associated X protein (Bax), and activation of caspase-3, caspase-9, and poly-ADP-ribose polymerase [9, 32]. Furthermore, anthracyclines activate, through oxidative stress, p38 mitogen-activated protein kinase (MAPK), which has a main role in the apoptotic process [33]. Moreover, DOX has been shown to mediate cardiomyocyte apoptosis through extrinsic pathway mediators such as death receptors (DRs) [34]. DOX might also decrease the expression of Mitofusin 2 (Mfn2), a mitochondrial GTPase fusion protein, to cause mitochondrial fragmentation and ROS generation, further causing cardiomyocyte apoptosis [35].

Calcium dysregulation is another well-known and established mechanism contributing to anthracycline-induced cardiotoxicity [36]. Anthracyclines might modulate the sarco/endoplasmic reticulum Ca21 ATPase (SERCA) present on sarcoplasmic reticulum (SR) and the sodium/potassium exchanger on sarcolemma [37, 38] while mitochondrial ROS generated from the exposure of cardiac cells to DOX might lead to an increase in cytosolic calcium levels. Increased levels of calcium is correlated with calcineurin-dependent activation of the nuclear factor of activated T-lymphocytes, which promotes cardiac cell death. In addition, anthracyclines may also alter adrenergic and adenylate cyclase function to trigger abnormalities in ${\mathrm{Ca}}^{2+}$ handling and therefore induce systolic ventricular dysfunction [21].

Recently, Wang *et al*. [25] found that the DOX-activated TRPA1 channel in cardiomyocytes could also cause cardiotoxicity by promoting endoplasmic reticle stress (ER) stress.

Narikawa *et al*. [39] demonstrated that DOX could increase the expression of metalloproteases, transforming growth factor-β, and collagen in human cardiac fibroblasts through phosphoinositide 3-kinase (PI3K)/Akt signaling pathway activation in order to produce an extracellular matrix imbalance, resulting in fibrosis and cardiac dysfunction.

Evidence about the effect of anthracyclines on autophagy regulation in cardiomyocytes is controversial [32]. It has been shown that DOX could stimulate autophagy through increased ratio of microtubule-associated proteins 1A/1B light chain 3-II and upregulated expression of p62, Beclin-1, by stimulating the expression of c-Jun N-terminal kinases and p70S6 kinase [32]. Furthermore, the inhibition of mechanistic target of rapamycin by DOX promotes autophagy [32].

**Fig. 1.**
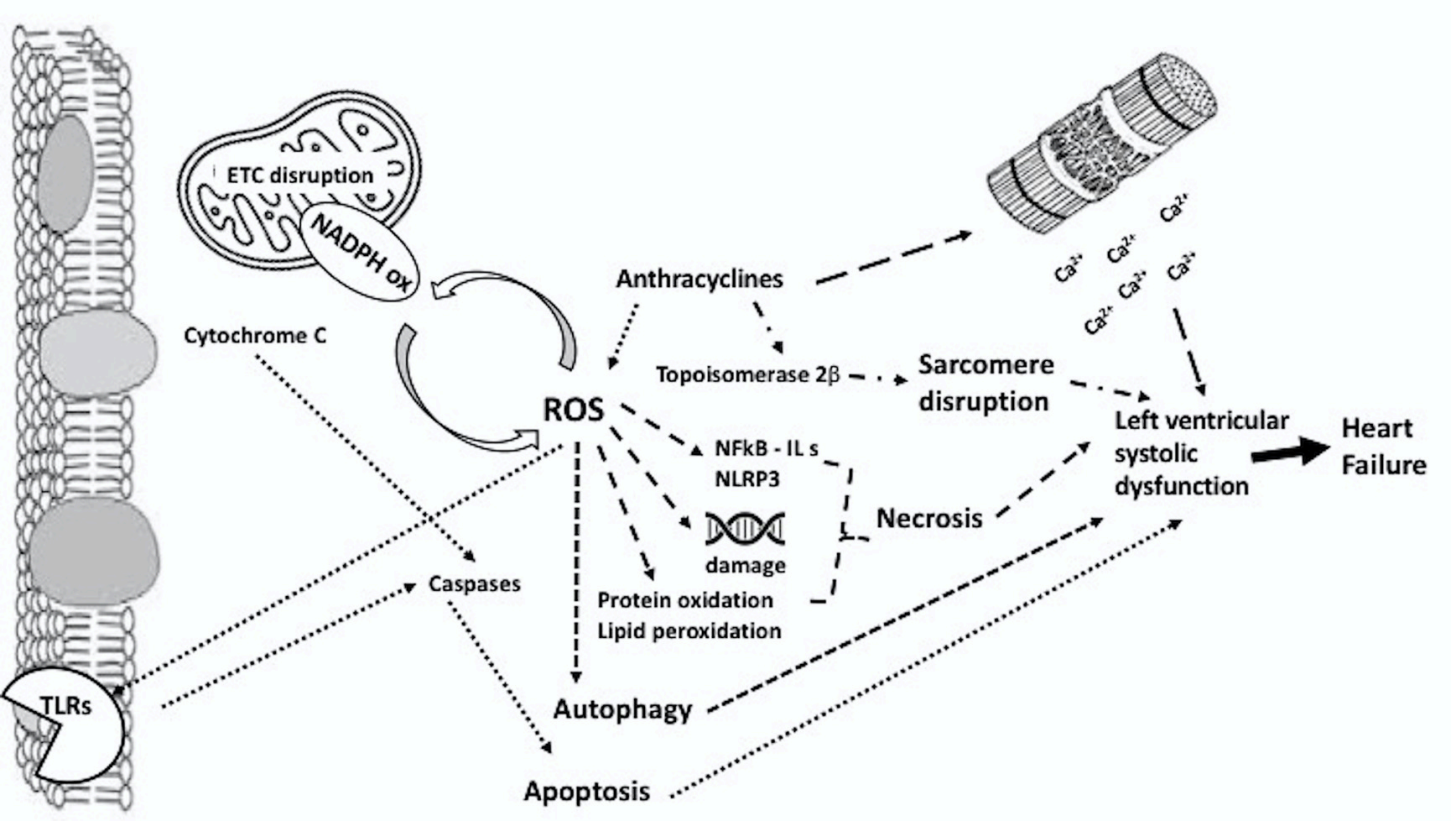
**Main molecular mechanisms of anthracyclines-induced cardiotoxicity**. Each arrow pattern, refers to a different molecular pathway. For more details, see the text. AC, anthracyclines; Ca, Calcium; ETC, electron transport chain; ILs, Interleukins; NADPH, Nicotinamide Adenine Dinucleotide Phosphate; NFkB, nuclear factor kappa-light-chain-enhancer of activated B cells; NLRP3, NLR family pyrin domain containing 3; ROS, reactive oxygen species; TLRs, Toll-like receptors.

Finally, anthracyclines may suppress protein synthesis by directly binding to DNA and may also induce sarcomere disruption, with the ensuing cardiac “sarcopenia” being typically associated to anthracycline-induced HF [40]. Cardiomyocytes are not the unique target of anthracycline toxicity, indeed endothelial cells, progenitor cells and fibroblasts in the heart, are also targets, contributing to a multifaced pathogenesis of anthracycline-induced cardiotoxicity. Increased arterial stiffness due to endothelial vascular damage caused by the alteration of the vascular extracellular matrix and by the interference with the endothelial regulation of vascular tone due to reduction of nitric oxide synthesis is also associated with anthracyclines. They may also increase the expression of cytokines leading to inflammation and vascular damage [41]. The main proposed pathogenetic mechanisms of anthracyclines-induced cardiotoxicity are summarized in Fig. 1.

## 5. Risk Stratification for Anthracyclines-Induced Cardiotoxicity

Previous epidemiological and observational studies have shown that specific risk factors in the clinical history of patients undergoing chemotherapy with anthracyclines may increase the chance to develop cardiotoxicity [16, 42, 43]. It is fundamental to recognize and, whenever possible, treat these conditions in order to prevent and to allow an early detection of anthracyclines-induced cardiotoxicity. A recent meta-analysis has shown that traditional cardiovascular risk factors, such as arterial hypertension (odds ratio (OR): 1.99; 95% confidence interval (CI): 1.43–2.76), diabetes mellitus (OR: 1.74; 95% CI: 1.11–2.74), and obesity (OR: 1.72; 95% CI: 1.13–2.61), are associated with an increased risk of cardiotoxicity. Tobacco smoke (OR: 1.62; 95% CI: 0.94–2.77) and hypercholesterolemia (OR: 1.48; 95% CI: 0.99–2.20) are less associated to cardiotoxicity [44]. Chronic kidney disease, pre-existing LV dysfunction, and pre-existing cardiovascular diseases, such as congestive HF, valvular heart disease, and ischemic cardiomyopathy, have been shown to increase the risk of cardiotoxicity [16]. Pharmacogenomics is emerging as a potential tool to help identify patients who are at higher risk for cardiotoxicity [45]. For example, Aminkeng *et al*. [46] highlighted that a nonsynonymous variant in Retinoic Acid Receptor Gamma (RARG) gene is highly associated with anthracyclines induced cardiotoxicity. Moreover, risk factors associated with cancer therapies, such as a previous or high dose of anthracyclines (≥250 mg/$m^{2}$ of DOX or equivalent), additional drugs, or radiotherapy, may also increase the risk of cardiotoxicity [47]. Recently, the Cardio-Oncology Study Group of the Heart Failure Association (HFA) of the European Society of Cardiology (ESC) in collaboration with the International Cardio-Oncology Society (ICOS) proposed a cardiovascular risk stratification system that can be applied in patients before starting therapy with anthracyclines. According to HFA-ICOS risk assessment, patients can be classified into low, moderate, high and very high risk [48]. Baseline cardiotoxicity risk assessment of patients undergoing to anthracycline treatment is summarized in Table 2, in accordance to the 2022 ESC guidelines on cardio-oncology [13]. Subsequent surveillance protocols depend on the baseline risk profile of each patient [13].

**Table 2.** **Baseline assessment of the risk of cardiotoxicity in patients undergoing to anthracycline treatment**.

Risk factors	Risk level
Congestive HF or cardiomyopathy	Very High
Coronary artery disease	High
LVEF reduction (<50%)	High
Age ≥80 years	High
Previous anthracycline-based chemotherapy	High
Previous left chest or mediastinum radiotherapy	High
Borderline LVEF (50–54%)	Medium (++)
Age 65–79 years	Medium (++)
Hypertension	Medium-low (+)
Diabetes	Medium-low (+)
Chronic kidney disease	Medium-low (+)
Previous non-anthracycline-based chemotherapy	Medium-low (+)
Current smoker or smoking history	Medium-low (+)
Obesity	Medium-low (+)
Elevated baseline troponin	Medium-low (+)
Elevated baseline BNP or NT-proBNP	Medium-low (+)

BNP, brain natriuretic peptide; HF, heart failure; LVEF, left ventricular ejection fraction; NT-proBNP, N-terminal prohormone of brain natriuretic peptide. “Very High Risk” patients: congestive HF or cardiomyopathy; “High Risk” patients: ≥5+ or any high-risk factors; “Medium Risk” patients: 2+ or 3+ or 4+ “Low Risk” patients: 1+ or no risk factors.

## 6. Effects of Anthracyclines at Different Ages

As stated previously, age is an important risk factor for anthracycline-induced cardiotoxicity [13]. Patients aged between 65–79 years are considered at medium-risk whilst patients aged ≥80 years are deemed high-risk [13]. Similarly, young patients have also a higher risk of developing anthracycline-induced cardio-toxicity [19] for several reasons. Sarosiek *et al*. [49] demonstrated that cardiac mitochondria in adult mice and humans are resistant to pro-apoptotic signaling while cardiac mitochondria in young individuals are primed for apoptosis, predisposing cells to death in response to toxic injuries. Additionally, children could have higher anthracycline levels in blood and tissues, which exacerbates adverse effects. In fact, anthracyclines have a lipophilic nature and children have an increased percentage of body fat [50]. Furthermore, anthracyclines, through interactions with topoisomerase, are known to target proliferating cells such as cardiac progenitor cells [51], which are most abundant in the neonatal period [52]. The loss of these cells, which have the capability to restore myocardium after injury [53, 54], could damage cardiac repair mechanisms and lead to Grinch syndrome [55], a form of cardiac remodeling characterized by decreased cardiac size that occurs in childhood cancer survivors treated with anthracycline. On the other hand, advanced age is a risk factor for anthracycline-induced cardiotoxicity due to a higher incidence and prevalence of hypertension, diabetes mellitus, preexisting cardiac diseases, and other comorbidities [56, 57]. Moreover, elderly individuals might have altered pharmacokinetics or pharmacodynamics of anthracyclines, which render them more vulnerable to adverse effects [58, 59]. Finally, an increasing prevalence of polypharmacy in the elderly predisposes this age group to an increased risk of toxicity [60, 61].

**Classification of Anthracycline-associated Cardiotoxicity**

Chemotherapy-associated cardiotoxicity can be divided into five main types:

Type 1: Cardiac dysfunction/cardiomyopathy/HF (cancer therapy related cardiac dysfunction CTRCD)

Type 2: Myocarditis

Type 3: Vascular toxicity

Type 4: Hypertension

Type 5: Arrhythmias and QTc prolongation [62].

**Fig. 2.**
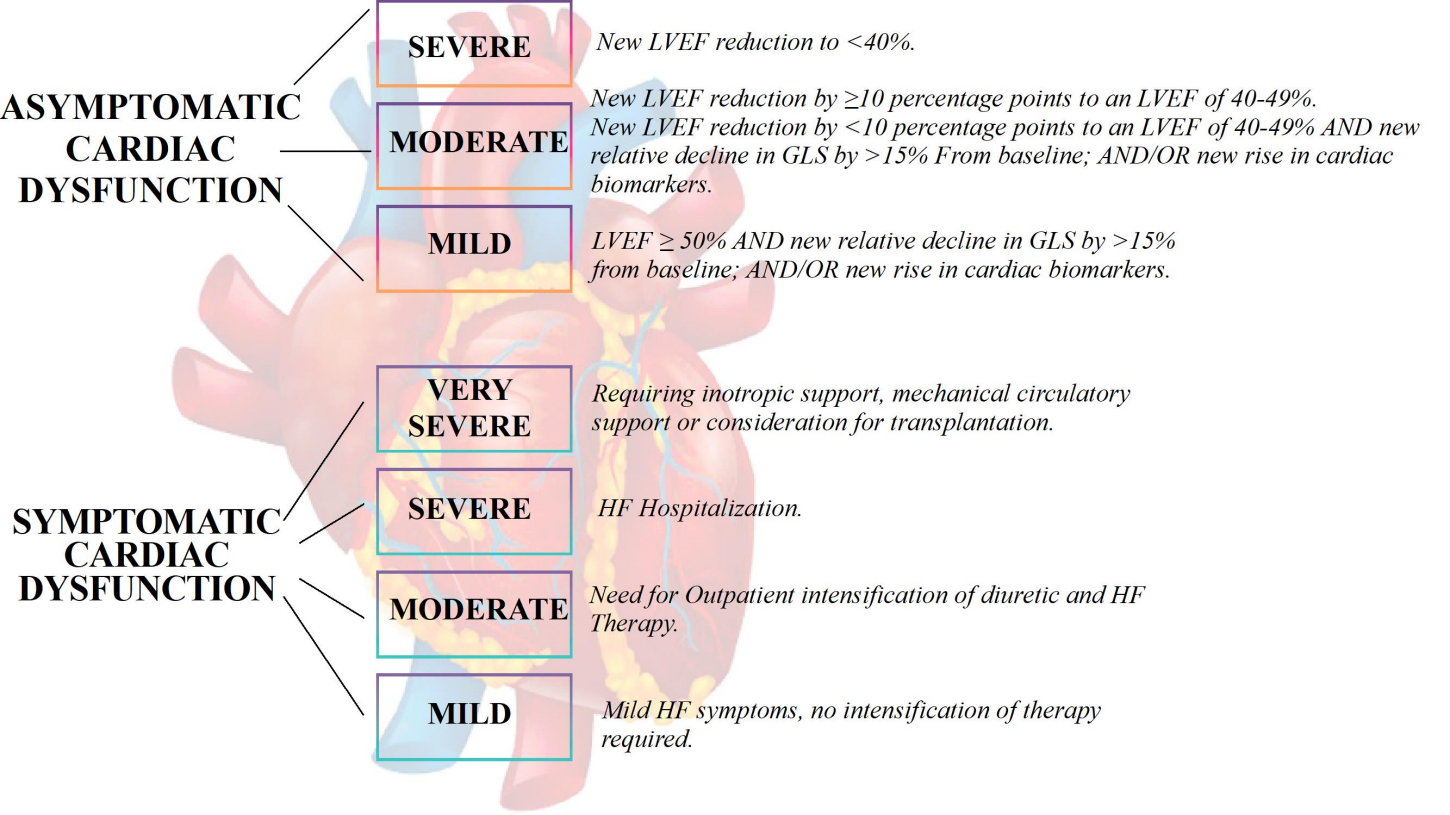
**Classification of Cancer Therapy Related Cardiac Dysfunction **. LVEF, Left Ventricular Ejection Fraction; GLS, Global Longitudinal Strain; HF, heart failure.

Anthracyclines are primarily associated with cardiac dysfunction (type 1 cardiotoxicity). According with the ICOS consensus statement, cardiac dysfunction is divided into symptomatic and asymptomatic [24]. Symptomatic systolic dysfunction is characterized by symptoms and signs of HF due to structural or functional heart damage. It is classified into very severe, severe, moderate, and mild based on the intensity of symptoms and the need for hospitalization. Asymptomatic cardiac dysfunction is defined as LV ejection fraction (LVEF) ≤50% and new relative decline in global longitudinal strain (GLS) >15% from baseline and/or new rise in cardiac biomarkers (troponin I/T >99th percentile, brain natriuretic peptide, BNP ≥35 pg/mL, NT-pro BNP ≥125 pg/mL) [62] (Fig. 2).

## 7. Multimodality Imaging Evaluation of Anthracyclines-Induced Cardiotoxicity

**Fig. 3.**
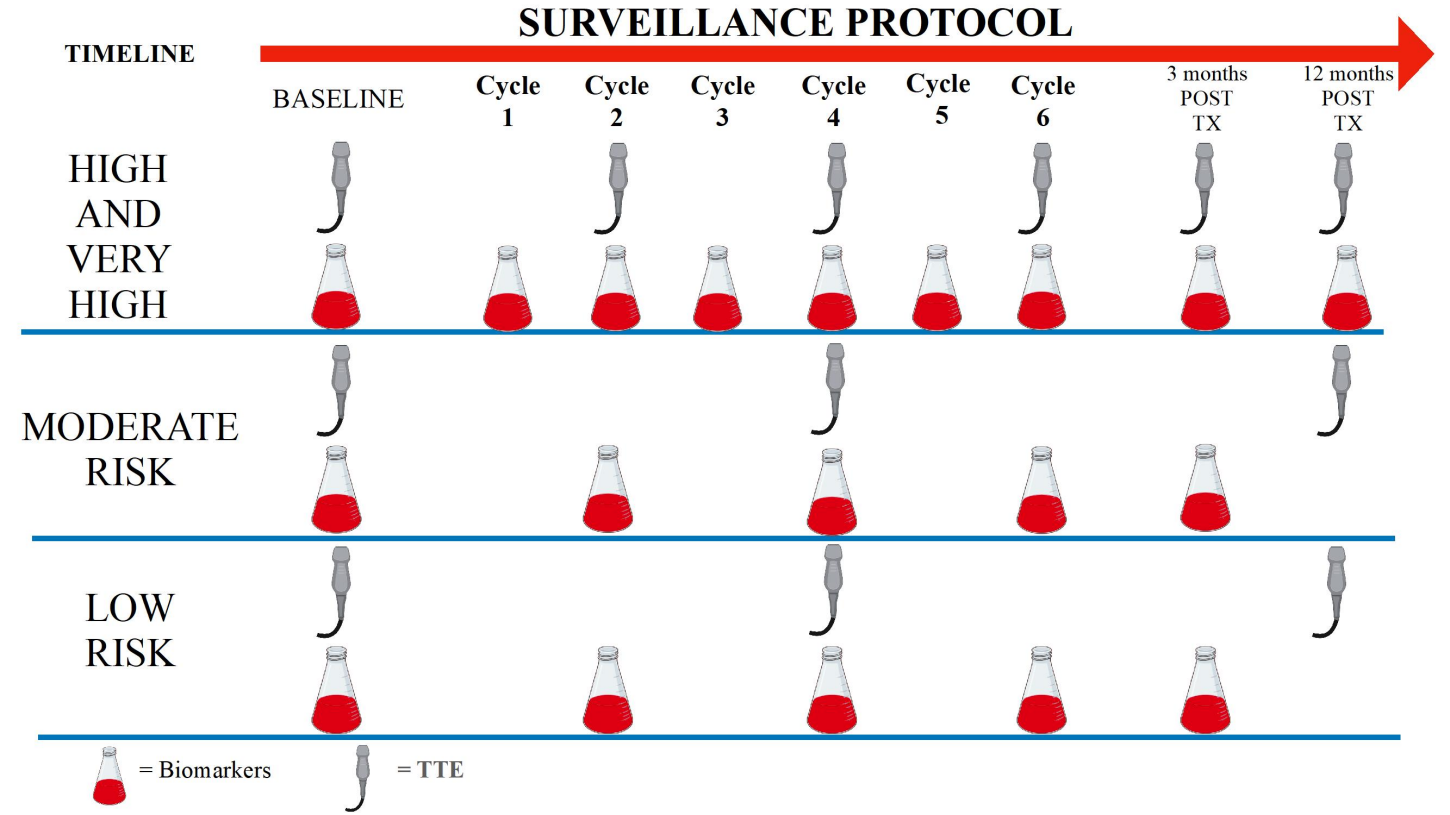
**Surveillance protocol during anthracycline treatment**. TX, Treatment; TTE, Trans Thoracic Echocardiography.

Over time, multiple surveillance protocols have been proposed, according to patients’ baseline risk of toxicity, to promptly diagnose anthracyclines-induced cardiotoxicity as to avoid the progression to HF. Most these protocols use repeated echocardiography and blood tests. The surveillance protocol recently proposed by the ESC is shown in Fig. 3 [13]. Due to its reproducibility, versatility, and availability, echocardiography appears to be the cornerstone method for the evaluation of patients affected by neoplasms who are candidates to chemotherapy [63]. Modified biplane Simpson’s technique [31] 2D echocardiography (2DE) has been the most widely used tool for the evaluation of ventricular contractility [31]. Despite this, it suffers from a series of limitations:

∙ LV geometric assumption

∙ Inadequate apex visualization

∙ Lack of consideration of subtle regional wall motion abnormalities

∙ Inherent variability of the measurement [64]

Compared to the 2D method, 3D echocardiography (3DE) allows more accurate volume measurements as it is not affected by geometric approximations and suffers less temporal variability and has a better intra-interobserver and test-retest variability [65]. For that reason, in agreement with the latest guidelines of the European Society of Cardiology, 3DE appears to be the method of choice for measuring the volumes and systolic function of the left ventricle [65]. 2DE also fails to detect small changes in LV contractility, underestimating the rate of mild asymptomatic cardiac dysfunction. The scientific interest has therefore focused on other parameters, such as those estimating myocardial deformation (strain and strain rate).

Strain refers to the patterns of myocardial contraction and relaxation that occur during each cardiac cycle. It encompasses radial, circumferential, and longitudinal strain. While evidence for the use of radial and circumferential strain is scarce, GLS is a parameter with high reproducibility and accuracy for early detection of subtle alterations in myocardial function that precede changes in LVEF [66].

Negishi *et al*. [67] evaluated women with breast cancer who underwent treatment with trastuzumab (46% of whom received anthracycline sequentially with trastuzumab) and found that a relative decrease of 11% in GLS was strongly associated with CTRCD. Similarly, Wang *et al*. [68] found that, in patients affected by diffuse large B-cell lymphomas and undergoing anthracycline treatment, a relative GLS decrease of 13.8% at the third month of chemotherapy was the best predictor of CTRCD, with a sensitivity of 75% and specificity of 91% (Table 3, Ref. [67, 68, 69, 70]). In accordance, the American Society of Echocardiography (ASE) and the European Association for Cardiovascular Imaging (EACVI) suggest that a relative decline in GLS >15% is likely to indicate subclinical LV dysfunction [63]. A recent meta-analysis found that also the absolute values of GLS can be used in the detection of CTRCD in those who did not perform a baseline echocardiography or in those patients in whom the baseline GLS is not performed [71]. However, there is currently lack of strong evidence to suggest a GLS-based cardioprotective approach (CPT) and a strain-guided management of follow-up for patients exposed to potentially cardiotoxic therapies [72]. In the Strain Surveillance of Chemotherapy for Improving Cardiovascular Outcomes (SUCCOUR) trial involving 331 patients, of which most had breast cancer, treated with anthracyclines, a CPT strategy based on GLS was compared to an approach based on LVEF. Despite its primary outcome not being reached, patients in the GLS-guided arm experienced less CTRCD compared to LVEF-guided arm (5.8% vs 13.7% in the EF (*p* = 0.022)) [72].

**Table 3.** **Prognostic values of Global longitudinal strain (GLS)**.

	Primary end point	Definition of CTRCD	INDEX	AUC	Sensitivity and Specificity
Wang *et al*. [68]	Early detection of CTRCD	LVEF reduction ≥10% to a value <53%	Relative GLS reduction of 13.8% at the third cycle of chemotherapy	0.826	75% and 91%
Negishi *et al*. [67]	Early detection of CTRCD	Symptomatic LVEF reduction of 5% or an asymptomatic 10% reduction to an LVEF of 55%	Relative GLS reduction of 11% at 6 months after the start of chemotherapy	0.84	65% and 94%
Gripp *et al*. [69]	Early detection of CTRCD	Symptomatic LVEF reduction of 5% or an asymptomatic 10% reduction to an LVEF of 55%	Relative GLS reduction of 14% at 3 months after the start of chemotherapy.	0.97	80% and 99%
Sawaya *et al*. [70]	Early detection of CTRCD	Reduction of LVEF ≥5% to <55% with symptoms of HF or an asymptomatic reduction of LVEF ≥10% to <55%	Relative GLS reduction of 11% at 3 months after the start of chemotherapy.		78% and 79%

AUC, area under the curve; CTRCD, cancer therapy related cardiac dysfunction; GLS, Global longitudinal strain HF, heart failure; LVEF, left ventricular ejection fraction.

Despite its reproducibility and better accuracy than echocardiography in the evaluation of cardiac volumes and function, cardiac magnetic resonance (MRI) is currently not routinely used [63]. It is particularly useful for the evaluation of cardiac masses and in case of technical difficulties in performing echocardiography [13]. Similarly, multigated acquisition (MUGA) has also a limited role, with it being recommended only when echocardiography is equivocal and MRI is not available [13].

## 8. Role of Biomarkers in Detecting Anthracyclines-Induced Cardiotoxicity

There is still a great debate on the use of biomarkers in the setting of CTRCD [73]. Even though, they can identify subclinical LV dysfunction, the evidence in favor of their routine use in the follow up of patients undergoing chemotherapy is scarce and mostly based on expert opinions [74]. Most of the available evidence involves the use of cardiac troponins (cTnT/I) and natriuretic peptides (NP), such as brain natriuretic peptide (BNP) and N-terminal prohormone of brain natriuretic peptide (NT-Pro BNP). cTnT/I are markers of myocardial injury and their role in the context of cardiac ischemic disease is well established [75]. In a study conducted on 703 patients with breast cancer undergoing anthracyclines-based chemotherapy, it was shown that an increase in Troponin I levels at 3 and 6 months was associated with an increased risk of LV systolic dysfunction [76]. Recently a meta-analysis conducted on 61 trials with 5691 patients investigated the predictive values of both cTnT/I and NP. They found that cTnT/I, but not NP, might be a useful screening marker for systolic dysfunction (negative predictive value of 93%) [77]. Furthermore, a combined diagnostic approach with cTnT/I and imaging (such as GLS) could increase its ability to predict systolic dysfunction [70]. Nevertheless, there is no conclusive evidence regarding the association between a rise in cTnT/I levels and the development of cardiotoxicity-related HF or cardiotoxicity-related mortality. While NP are a cornerstone in the diagnosis of HF [78], their role as a predictive tool for cardiotoxicity is less clear. Since NP are strongly related to a patient’s fluid volume status, their diagnostic power could be limited [74]. Rug̈er *et al*. [79] have shown that levels of NT-pro-BNP measured at week 6 of anthracycline-regimen in 853 patients with breast cancer was significantly associated with the development of cardiotoxicity (OR: 1.03; 95% CI: 1.008–1.055; *p* = 0.01). Most guidelines currently recommend measuring both biomarkers at baseline and repeatedly during a chemotherapy regimen, in relation to the baseline risk of cardiovascular toxicity. However, uncertainties about the correct timing still persist [13].

## 9. Prevention and Treatment of Anthracyclines-Induced Cardiotoxicity

Recommendations regarding preventive measures are based on the baseline risk of anthracycline-related cardiotoxicity. In patients at high- and very high-risk pf cardiotoxicity or in those who undergo high doses of anthracyclines (i.e., DOX >300 mg/$m^{2}$), it is recommended to use specific cancer-related therapies such as dexrazoxane. The cardioprotective mechanism of dexrazoxane is not fully understood, but it has been attributed to its strong iron chelating properties that could reduce the production of ROS during anthracycline therapy [80]. Dexrazoxane was also shown to downregulate topoisomerase 2β and prevent the formation of a complex between topoisomerase 2β and anthracyclines [81]. However, concerns about the safety of dexrazoxane have been raised [50]. A systematic review suggested that patients treated with dexrazoxane could have a low response rate to anthracycline [82]. However, an updated version of the same study failed to confirm its findings [83]. More recently, a meta-analysis of 13 randomized clinical trials not only confirmed the cardioprotective effect of dexrazoxane when added to anthracycline-based chemotherapy (risk ratio (RR): 0.22, 95% CI: 0.11–0.43), but also indicated that dexrazoxane does not affect the anticancer properties of anthracyclines since there was no difference in tumor response rate in the dexrazoxane group (RR: 0.91, 95% CI: 0.79–1.04) [84]. Hence, dexrazoxane is currently approved by The Food and Drug Administration (FDA) and by the European Medicine Agency (EMA) to reduce the cardiotoxicity effect of anthracycline in women with metastatic or advanced breast cancer who have received a cumulative DOX dose of 300 mg/$m^{2}$ and who will continue to receive doxorubicin (DOXO) therapy to maintain tumor control. Moreover, in 2017 EMA removed the contraindication for children and adolescents treated with high cumulative doses of anthracyclines [83, 84, 85].

Alternatively, liposomal preparations of DOX are used to reduce anthracycline toxicity [86] as they block their entry into cardiac cells, thus limiting their cardiotoxic effect.

Current guidelines recommend starting a preventive therapeutic strategy with beta blockers (BB), angiotensin converting enzyme inhibitors (ACEI) and statins in patients with high-/very high-risk of developing cardiotoxicity and in patients with mild/moderate asymptomatic systolic dysfunction. Neurohormonal therapy may play a crucial role in preventing cardiotoxicity. Data pointing to a positive effect of ACEi and BB in preventing the decrease in LVEF are summarized in Table 4 (Ref. [87, 88, 89, 90, 91]). These findings are consistent with a recent meta-analysis of 17 trials with a total of 1984 patients with a follow-up ranging from 4 months to 2 years [92]. However, it is currently debated whether the beneficial effect of neurohormonal therapy might translate into improved clinical outcomes. It is also interesting to note that in this large meta-analysis the absolute improvement in terms of LVEF assessed by 2DE was only 5%, i.e., that is within the range of interest variability of the measurement.

**Table 4.** **Neurohormonal therapy to prevent anthracycline cardiotoxicity**.

	Drugs used	Type of cancer	Inclusion criteria	Primary endpoint	Results vs controls
Janbabai *et al*. [87]	Enalapril 5 mg bid	Breast Cancer	Normal LVEF; Normal troponin level	6 months LVEF change from baseline	59.61% ± 5.70 vs 46.31% ± 7.04 (*p *< 0.001)
Bosch *et al*. [88]	Enalapril 2.5 bid + Carvedilol 6.25 bid	Hematological	Normal LVEF + Normal troponin level	6 months LVEF change from baseline	–0.17 (–2.41 to 3.13) vs –3.04 (–6.01 to 0.11) (*p* = 0.04)
		Malignancies			
Kalay *et al*. [89]	Carvedilol 12.5 mg od	Breast cancer and Lymphoma	Normal LVEF; Normal troponin level	6 months LVEF change from baseline	68.9% vs 52.3% (*p *< 0.001)
Cardinale *et al*. [90]	Enalapril 20 mg od	Breast and Hematological malignancies	Increased Troponin level; Normal LVEF	Occurrence of cardiotoxicity	0 (0%) vs 25 (43%) (*p *< 0.001)
Avila *et al*. [91]	Carvedilol from 3.125 mg bid to 25 mg bid	Breast cancer	Normal LVEF	Prevention of a 10% reduction in LVEF	14 (14.5%) vs 13 (13.5%), *p* = 1

LVEF, Left Ventricular Ejection Fraction.

Statins, among their pleiotropic effects, can also reduce ROS generation and can inhibit topoisomerase II. Since both these mechanisms are involved in anthracycline-related cardiotoxicity, a beneficial effect of statins has been hypothesized [93]. Nabati *et al*. [94] evaluated the effect of rosuvastatin 20 mg od in the prevention of anthracycline-related cardiotoxicity during a 6 months follow-up, with rosuvastatin having prevented a 2DE estimated drop in LVEF in the intervention group. However, there was no difference between the two groups of patients with regards to GLS. The available evidence investigating the role of statins in preventing anthracycline-related cardiotoxicity is summarized in Table 5 (Ref. [94, 95]).

**Table 5.** **Evidence on the protective role of statins to prevent anthracycline cardiotoxicity**.

	Drugs used	Type of cancer	Inclusion criteria	Primary endpoint	Results: Intervention vs control
Nabati *et al*. [94]	Rosuvastatin 20 mg od	Breast cancer	Normal LVEF	Changes in the LVEF	53.54% vs 49.95% (*p* = 0.015)
Acar *et al*. [95]	Atorvastatin 40 mg od	Hematologic disorders	Normal LVEF	Patients with LVEF <50% after 6 months	1 vs 5 (*p* = 0.18)

LVEF, Left Ventricular Ejection Fraction.

Sacubitril/Valsartan and sodium-glucose co-transporter-2 (SGLT2i) are mainstays for the treatment of HF with reduced EF (HFrEF), with their efficacy having been shown in different trials and in both acute and chronic settings [96, 97, 98]. However, history of chemotherapy-induced cardiomyopathy over 12 months was an exclusion criterion in the main trials for sacubitril/valsartan, such as the Angiotensin–Neprilysin Inhibition versus Enalapril in Heart Failure trial (PARADIGM-HF) trial [97]. Likewise, patients with active malignancy required treatment were excluded in the Dapagliflozin in Patients with Heart Failure and Reduced Ejection Fraction (DAPA-HF) trial. Therefore, solid indications regarding the use of sacubitril/valsartan and SGLT2i for this purpose is lacking. Recently, Garcia *et al*. [99] provided evidence on the efficacy and safety of Sacubitril/Valsartan for CTRCD and HFrEF (LVEF <40%) in a retrospective cohort of 67 patients. Most of these patients were women with breast cancer, mainly treated with anthracycline (70%) and in median follow-up of 4.6 month. Patients treated with Sacubitril/Valsartan showed a significant improvement of LVEF from baseline as well as reversed remodeling. There was also an improvement in New York Heart Association (NYHA) functional class (NYHA functional class 2.2 ± 0.6 vs 1.6 ± 0.6). In terms of safety endpoints, there were no differences between basal and follow-up levels of serum creatinine or potassium. Evidence regarding the effect of gliflozins on anthracycline-related cardiac toxicity is currently limited, despite different studies having proven their positive effect in DOX-induced cardiomyopathy in animal models [100]. A recent study Gongora *et al*. [101] evaluated the cardioprotective role of SGLT2i in a retrospective cohort of patients with diabetes mellitus on treatment with SGLT2 and receiving anthracycline-based chemotherapy. The primary endpoint was a composite of HF incidence, HF admission and cardiomyopathy defined as a 10% > decline in LVEF. Primary outcomes were lower in patients treated with SGLT2 compared to the control group not on SGLT2 (3% vs 20%; *p* = 0.025) while the SGLT2 group experienced fewer HF admission and cardiac dysfunction. Moreover, the SGLT2 group had an improvement in the survival rate. Further studies are therefore needed to confirm the cardioprotective effect of SGLT2 in patients undergoing treatment with anthracyclines.

**Fig. 4.**
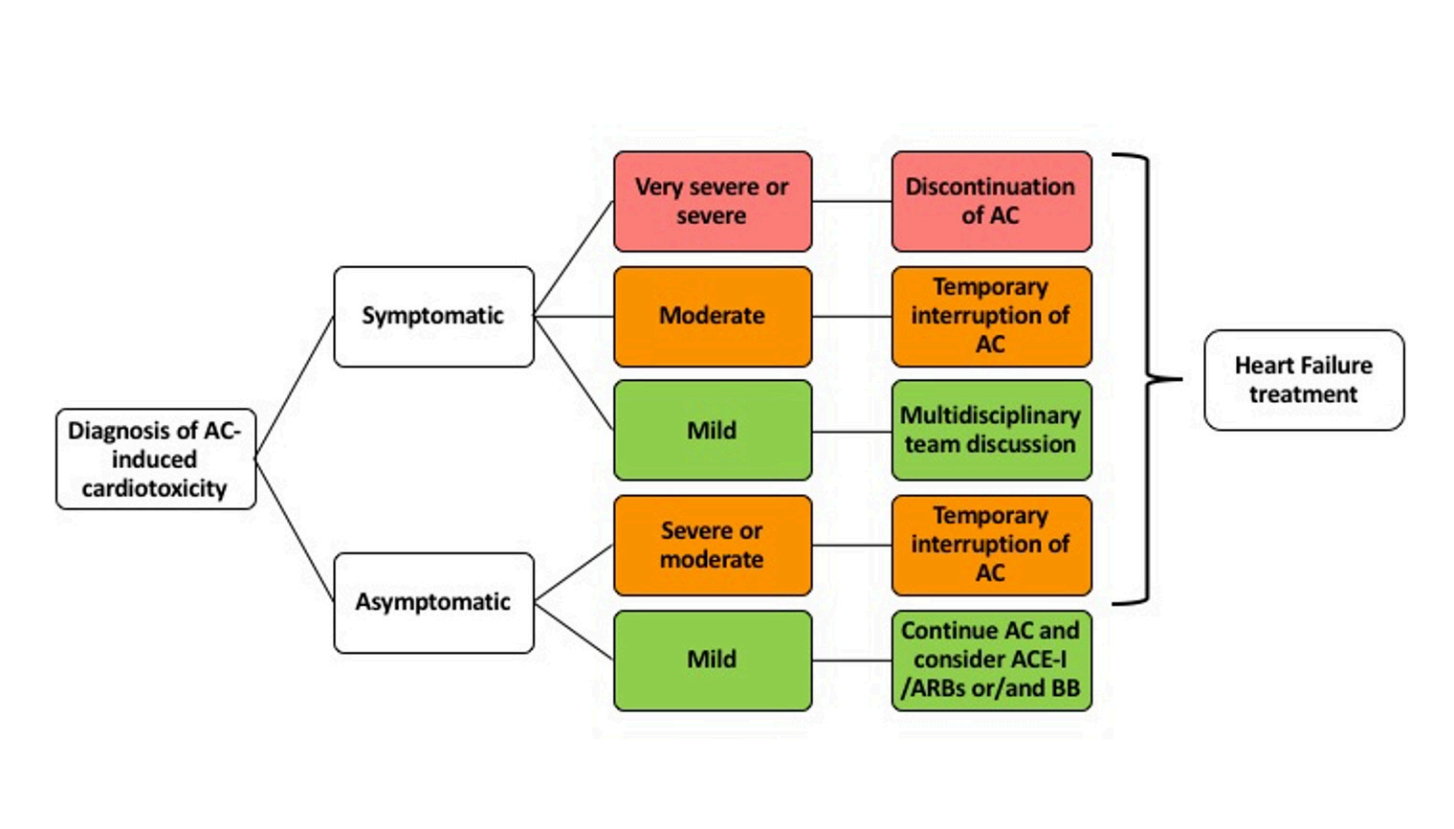
**Management of patients with anthracycline-induced cardiotoxicity**. AC, Anthracyclines; ACE-I, Angiotensin-Converting Enzyme Inhibitors; ARBs, Angiotensin Receptor Blockers; BB, Beta Blockers.

The management of patients affected by anthracycline-induced cardiotoxicity is summarized in Fig. 4, in accordance with the 2022 ESC guidelines on Cardio-Oncology [1]. With regards to the treatment of established anthracycline-induced cardiotoxicity, it is recommended to suspend chemotherapy and start cardiovascular therapy when symptoms related to HF appear, in accordance with the 2021 ESC guidelines on the management of HF [1].

## 10. Conclusions

Cardiotoxicity is a potentially troublesome adverse effect of anthracycline-based chemotherapies since they may cause LV systolic dysfunction followed by HFrEF, which tends to be permanent. It is thus of great importance to assess the risk of cardiotoxicity before anthracyclines therapy, to structure a follow-up plan that is tailored on individual patient’s risk. Despite there being a general consensus on the role of echocardiography in diagnosing anthracycline-related cardiotoxicity, the optimal timeframe to perform it and the optimal parameters to be evaluated for the diagnosis are still matter of debate. Biomarkers such as cTnI/T and NP have proved to have a good negative predictive value for anthracycline-related cardiotoxicity and as such, most recent guidelines recommend their serial measurement during follow-up. However, convincing evidence about ideal cut-off values, in terms of reliability, and definitive recommendations regarding its timing are lacking. Since most cardiotoxicity is early chronic (within 1 year from the start of anthracyclines), current guidelines recommend a strict follow up during the first year for patients at high- and very high-risk of cardiotoxicity along with the introduction of an ACEi/ Angiotensin Receptor Blockers (ARB) plus BB treatment regimen. Nevertheless, recommendations differ significantly between international guidelines. Due to cardiotoxicity being usually permanent, a deeper knowledge of the molecular pathways of action of anthracyclines and their effects on the cardiovascular system is crucial. Hopefully this might help minimizing their negative impact on heart and vessels and to develop more effective preventive strategies and therapeutic options for anthracycline-related cardiotoxicity. These are essential steps that would translate in a better survival, limited life-saving chemotherapy drug discontinuation, and better prognosis for patients undergoing anthracycline-based chemotherapies.
